# Unusual circumstance for craniopharyngioma discovery on meningoencephalitis: a pediatric case report

**DOI:** 10.1186/s12887-023-03930-5

**Published:** 2023-03-22

**Authors:** Jihann Oozeerally, Lionel Berthomieu, Anne-Isabelle Bertozzi, Bastien Estublier, Isabelle Oliver, Aurore Siegfried, Pierre Antherieu, Emilie Thene, Thibaut Jamme, Thierry Levade, Annick Sevely, Camille Brehin, Eloïse Baudou

**Affiliations:** 1 Service de Neurologie Pédiatrique, Hôpital Des Enfants, CHU Toulouse, 330 Avenue de Grande Bretagne, 31059 Toulouse, France; 2 Service de Réanimation Pédiatrique, Hôpital Des Enfants, CHU Toulouse, Toulouse, France; 3 Service d’Hémato-Oncologie, Hôpital Des Enfants, CHU Toulouse, Toulouse, France; 4 Service d’Endocrinologie Pédiatrique, Hôpital Des Enfants, CHU Toulouse, Toulouse, France; 5 Service d’Anatomopathologie, CHU Toulouse, Toulouse, France; 6 Service de Neurochirurgie, Hôpital Pierre Paul Riquet, CHU Toulouse, Toulouse, France; 7 Laboratoire de Biochimie, CHU Toulouse, Toulouse, France; 8 Service de Neuroimagerie, Hôpital Pierre Paul Riquet, CHU Toulouse, Toulouse, France; 9 Service d’Infectiologie et Urgences Pédiatriques, Hôpital Des Enfants, CHU Toulouse, Toulouse, France

**Keywords:** Craniopharyngioma, Chemical meningitis, Cholesterol, Children, Case report

## Abstract

**Background:**

Craniopharyngioma is a rare condition in children, but it is the most frequent tumor that occurs in the hypothalamic pituitary region. Chemical meningitis has been described as an uncommon postoperative complication, but no chemical meningitis due to a spontaneous rupture leading to craniopharyngioma diagnosis in children has been reported.

**Case presentation:**

This is a case of a 13-year-old boy presenting with fever, vomiting and headache for two days. The CT scan revealed a suprasellar lesion, and lumbar puncture showed aseptic meningitis. The cerebral MRI suggested a craniopharyngioma and the cerebrospinal fluid cholesterol concentration was abnormally high. A thorough medical history indicated some visual disturbance, which improved at the onset of meningitis, and an inflection of the growth curve. The anatomopathological analysis of the tumor confirmed the diagnosis of craniopharyngioma.

**Conclusions:**

This case is the first to report the discovery of a craniopharyngioma with meningoencephalitis caused by the rupture of a craniopharyngioma cyst in a child.

Diagnosis was facilitated by determining the cholesterol level in the cerebrospinal fluid, as well as fine anamnesis to identify visual and growth disturbances.

## Background

Craniopharyngioma (CP) is the most common non-glial intracranial pediatric tumor and represents approximately 10% of pediatric brain tumors. The peak incidence in children is between 5 and 15 years of age. CP is a partly cystic embryonic malformation that occurs in the sellar/parasellar region. Its diagnosis is often based on intracranial hypertensive symptoms, typically headaches, nausea and vomiting, visual or endocrine disturbances such as growth delay or diabetes insipidus caused by tumor extension.

There are two histological subtypes: adamantinomatous (ACPs) and papillary (PCPs) [1]. The ACP histological type with cyst is the most common in children and adolescents. During embryogenesis, Rathke’s pouch involution default with secondary proliferation of the embryonic cells around the craniopharyngeal duct, lead to the formation of a craniopharyngioma. Somatic *CTNNB1* mutations can be responsible for ACP development. ACP consists of a solid part characterized by dense nodules and squamous epithelial trabeculae bordered by a palisade of columnar epithelium and a cystic part that contains a yellow–brown, cholesterol-rich fluid [2].

If the content of a cyst spills into the subarachnoid space, meningeal signs can be observed. A few pediatric cases have been reported after surgery (Yasumoto et al*.* [3], Kumar et al. [4], Shinohara et al. [5], Patnaik et al. [6]) but spontaneous rupture is less common. However, pediatric cases of cystic rupture causing meningitis syndrome have already been reported but only in children with a prior diagnosis of CP.

## Case presentation

A 13-year-old boy with no medical history presented at pediatric emergency department of our hospital with complaints of episodes of severe headache associated with vomiting, which had been ongoing for two days. The next day, he was restless and had a fever. No other symptoms were reported by the family when the first medical history was taken. The Glasgow score was approximately 11–12/15. His body temperature was 38.1ºC, his blood pressure and heart rate were respectively 110/76 mmHg and 105 bpm. On clinical examination, confusion and a neck stiffness were found but no focal neurological deficit.

**Fig. 1:**
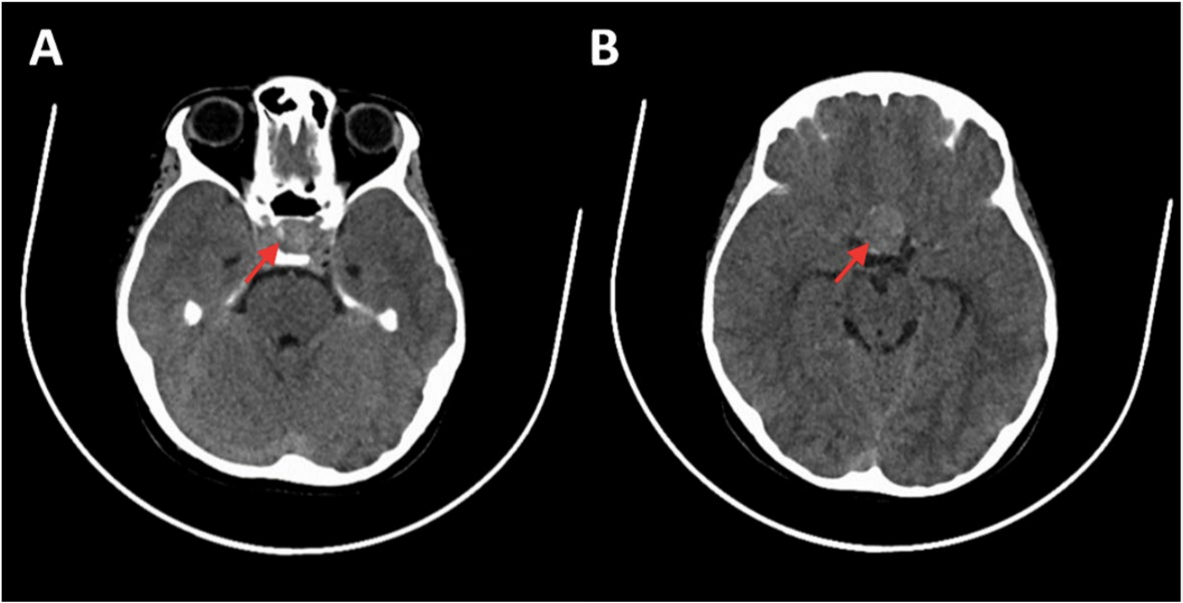
Cerebral tomodensitometry showing a calcified intra-(**A**) and suprasellar (**B**) lesion

Because of his confusion, he underwent a non-contrast enhanced CT scan, which revealed a heterogenous nodular lesion, spontaneously hyperdense, in the intra- and suprasellar regions, with minor calcification in the inferior area. There was no evidence of other lesions nor any signs of compression (Fig. 1).

The lumbar puncture revealed turbid cerebral spinal fluid (CSF). CSF showed pleocytosis with predominant neutrophils (90%), increased proteins (.,73 g/L) and decreased glucose (1.69 mmol/L), with a CSF glucose/blood glucose ratio < 0.5; chloride concentration was normal. Gram staining was negative.

Blood tests revealed hyponatremia (Na: 128 mmol/L) and a moderate inflammatory syndrome (CRP: 22.9 mg/L, white blood cells 9.600 G/L)); the urine sodium level was elevated. Hyponatremia was attributed to inappropriate secretion of the natriuretic factor caused by cerebral distress rather than inappropriate antidiuretic hormone secretion.

This clinical presentation led to treatment for bacterial meningitis. The patient was admitted to the intensive care unit and received intravenous cefotaxime for 7 days, gentamycin for 2 days, and corticosteroids for 4 days.

**Fig. 2:**
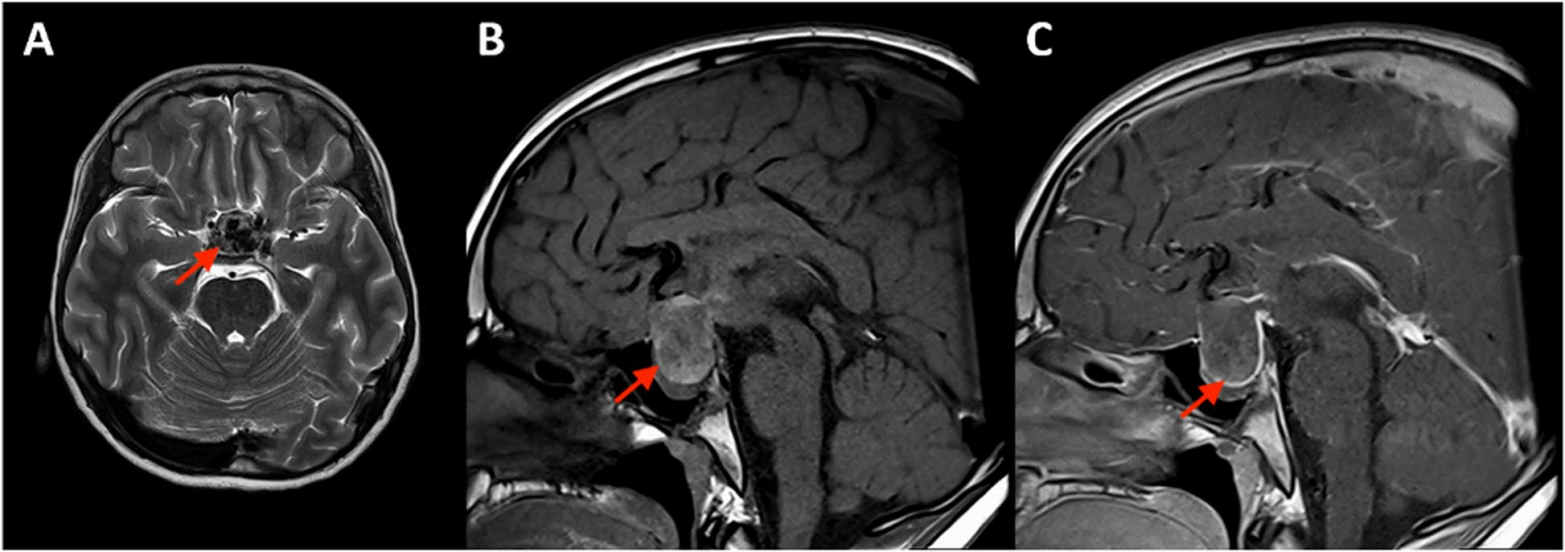
Cerebral MRI showing a cystic tumor in the intra- and suprasellar region, hypointense in the axial T2-weighted image (**A**), spontaneously hyperintense in the sagittal T1-weighted image (**B**) with an enhancement of the cyst wall after gadolinium injection (**C**)

The CSF bacterial culture as well as the blood culture were negative. PCR tests on CSF found no viral (HSV, VZV, HHV6 and enterovirus) infection. The MRI showed a cystic sellar and suprasellar lesion with heterogenous content. This lesion was characterized by hypointensities on T2-weighted images, suggesting the presence of calcifications, and hyperintensities in T1-weighted images corresponding to a high protein content. The optic chiasma was repressed with bilateral edema of the retrochiasmatic disks. The pituitary stalk was also compressed (Fig. 2). Together, the results of the CT scan and the MRI were evocative of CP. No spectroscopy was done.

The CSF cholesterol concentration was measured. The first determination using an enzymatic (cholesterol oxidase) method on a routine clinical chemistry automaton (Cobas 8000 Roche) indicated a value of 0.19 mmol/L. The second determination by gas chromatography combined with mass spectrometry gave a concentration of 0.14 mmol/L (a reference value of 0.012 mmol/L was obtained on a pool of CSF samples derived from controls subjects) (Table 1). Hence, the CSF cholesterol concentration in our patient was comparable to that previously reported in patients with CP.

**Table 1:** CSF sterol concentrations

	Patient	Controls (mean level)
Cholesterol (µmol/L)	140.5	12.4
7-dehydrocholesterol (µmol/L)	< 0.625	< 0.625
8-dehydrocholesterol (µmol/L)	< 0.3125	< 0.3125
Lathosterol (µmol/L)	83.6	< 0.625

Retrospectively, the medical history of this patient was suggestive of ophthalmological and endocrine disorders, which are classic symptoms of CP in children.

**Fig. 3:**
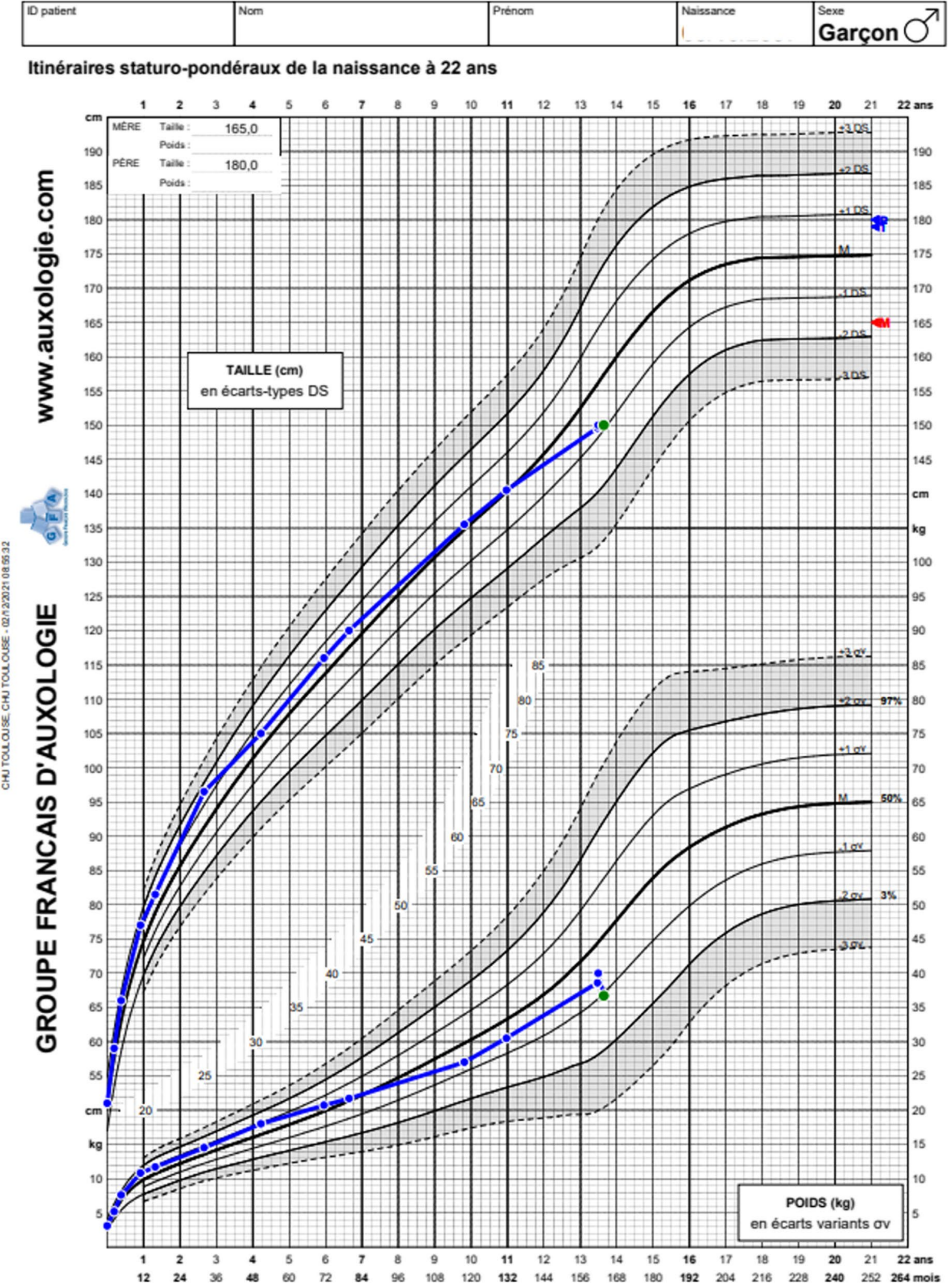
Growth chart showing a slowdown in growth from the age of 11. Upper part: size; lower part: weight

Analysis of his growth chart shows a slowdown in growth at around the age of 11 (Fig. 3).

Moreover, he had presented with vision disturbances a few weeks before hospitalization and which were alleviated concomitantly to the onset of headache. The ophthalmological assessment after this episode, including visual field testing, slit lamp and retinal examination and intraocular pressure measurement, was normal.

Hormone screening tests revealed deficits in Growth Hormone (IGF1: 123 ng/mL (-2.15 SD)) and, Thyroid Stimulating Hormone (TSH: 0.81 µIU/mL (0.27–4.2), T4: 14 pg/mL (9.3–17), T3 1.5 pg/mL (2–4.4), while other parameters were within the normal range (ACTH: 12 pg/mL (7.2–63.3), Cortisol: 3.51 µg/dL; FSH: 5.1 IU/L, LH: 2.7 IU/L, Testosterone: 1.18 nmol/L; Prolactin: 239 µIU/mL (68–324)). Hormonal replacement therapy with thyroxine was initiated. LH and FSH were normal, but the testosterone level was relatively low compared to the Tanner status (P3G3). Because of the corticosteroid therapy, cortisone levels could not be interpreted and a distant control test was normal.

The patient underwent surgery two months after the diagnosis. No fluid was withdrawn from the cyst. The cyst wall was described as relatively thick. Its content was consistent with long-standing hematoma. The days after surgery were uneventful.

The anatomopathological examination showed the presence of a mostly necrotic substance, with exceptional epithelial cells stained by AE1AE3 immunochemistry, normal hypophyseal structure and inflammatory cells. *BRAF*-V600E and beta-catenin gene mutations were absent and the NGS was negative. CP with necrotic changes was the principal hypothesis.

Post-operative imaging showed no evidence of residual tumor and; clinical and MRI follow-up was initiated.

## Discussion and conclusions

While chemical meningitis or CP symptom improvement has been largely reported after surgery, spontaneous rupture of CP is a rare condition in children [7]. In all the cases described in the literature except for one [8], CP was diagnosed weeks or months before rupture. Only in one case did the rupture of the cyst lead to the diagnosis of CP [8]. However, unlike this case, the rupture occurred in the brain parenchyma leading to a brain edema with headache and vomiting. Six additional cases of spontaneous rupture of CP in children have been reported, but all occurred in patients already diagnosed with CP. Two were asymptomatic and were diagnosed during imaging follow-up (Yasumoto et al*.* [3], Kumar et al. [4]), and two presented with febrile meningism [5, 6]. Characteristics of patients at cyst rupture and characteristics of the rupture are reported on Table 2.

**Table 2:** Pediatrics cases of spontaneous rupture of CP cyst in the literature

Study	Age (years)/ Sex	Diagnosis of CP before rupture	Rupture localization	Revealing symptom of rupture	CSF findings	
					Cholesterol crystals	Cholesterol concentration
Okamoto et al., 1985 [9]	17, M	Yes	Subarachnoid space	Alleviation of headache	Present	Not done
Satoh et al., [10]	17, M	Yes	Subarachnoid space	Alleviation of headache	Present	Not elevated
Shinohara et al., [5]	10, F	Yes	Subarachnoid space	Fever Headache Visual disturbance lassitude	Not found	Not done
Yasumoto et al. 2008 [3]	4, F	Yes	Subarachnoid space	Imaging follow-up	Not done	Not done
Kumar et al., [4]	13, M	Yes	Subarachnoid space	Imaging follow-up	Not done	Not done
Patnaik et al. [6]	13, F	Yes	Subarachnoid space	Fever Menigism Altered sensorium	Present	7 mg
Rajan et al. [8]	12, F	No	Intra-parenchymal: brain edema	Aggravation of headache vomiting	Note done	Not done
**Present case**	**13, M**	**No**	**Subarachnoid space**	**Alleviation of headache** **Fever** **Meningism**	**Present**	**0.14 mmol/L**

*CP* Craniopharyngioma, *CSF* Cerebral spinal fluid

The pathophysiology of this chemical meningitis is inflammation of the meninges caused by the release of the cyst cholesterol into the subarachnoid space. Diagnosis of a CP cyst rupture can be guided by CSF analysis revealing the presence of cholesterol crystals. CP was first described in 1934 by Weber et al. [11] as a cholesterol tumor of the pituitary body. Cholesterol crystals behave as irritants that trigger inflammation of the meninges [12]. In the asymptomatic spontaneous rupture of CP, the cystic portion might be too small or cholesterol levels too low in CSF to provoke symptoms [13]. Only in one case was, the cholesterol level determined and found to be unusually high [6]. Determination of CSF cholesterol is not a routine test and must be specifically requested. There were no established reference values to compare the results to in the case. Dedicated analysis was conducted by comparing the cholesterol content of the patient’s CSF to that of a pool of several control subjects with no CP (Table 1).

However, other tumor types can rupture and cause chemical meningitis. Garg et al. [14] described a patient with meningitis presenting good initial response to antibiotics and steroids but which deteriorated when steroids were discontinued [14]. The brain MRI and the spine MRI showed the presence of ruptured teratoma in the lumbo-sacral region. Fat droplets are described on the MRI and fat intensity signal reported. The diagnosis was confirmed by histopathology. In a literature review, Lunardi et al. [15] refer to nine cases of chemical meningitis in children due to the spontaneous rupture of a cranial or spinal tumor cyst [16]. The types of tumors reported were some dermoids, epidermoids, and CP and one glioma, two ependymomas and one teratoma.

Brain imaging plays a key role in orienting the diagnosis of CP. However, this exam had to be done at the optimal time in relation to the clinical signs of meningitis exhibited by the patient. Following the European guidelines (ESCMID guidelines 2016), it is strongly recommended to perform cranial imaging before lumbar puncture in patients with focal neurologic deficits (excluding cranial nerve palsy), new-onset seizures, severely altered mental status (Glasgow Coma Scale score < 10) or a severely immunocompromised status. In patients lacking these characteristics, cranial imaging before lumbar puncture is not recommended because it may lead to a delay in antibiotic treatment associated with a poor outcome. In this case report, there was no indication for emergency brain imaging according to these guidelines. Antibiotic treatment was urgently required due to the combination of clinical and biological signs of bacterial meningitis. However, brain imaging was important, but only once the results of the lumbar puncture ruled out bacterial and viral meningitis, in order to explore aseptic meningitis. Indeed, CP brain imaging characteristics allows to orient the diagnosis. Brain TDM showed intra and/or suprasellar lesion with heterogeneous density with cystic and solid components. The cystic component was hypodense but not as much as the CSF, and the solid component was isodense as brain parenchyma. Approximately 90% of the lesions contained calcifications located peripherally, which oriented the diagnosis. Dilatation of lateral ventricles could be associated with the compression of the third ventricle by the tumor. Brain MRI showed cystic sellar and suprasellar lesions with heterogenous content. The solid portion showed hypointensity on T1-weighted images enhanced with by contrast injection and was hyperintense on T2-weighted images. The cyst showed hyperintensities on T1-weighted images corresponding to a high protein content. Indirect signs of optic chiasma and pituitary compression could be seen. Based on MRI, the contours of the lesion and its relationship with adjacent structures could be defined for surgery. The diagnosis will be definitively confirmed after surgery by the anatomopathological analysis of the tumor.

Finally, the clinical history could identify signs that most commonly lead to the diagnosis of CP. These are ophthalmological and endocrinological signs respectively caused by optic chiasma and pituitary gland or pituitary axis compression. The visual disturbance described by the patient earlier in his medical history had disappeared immediately at the onset of headaches and, unfortunately had not been documented. A visual exam performed afterwards was normal. Rupture of the cyst could have led to a decrease in the tumor size which caused a rapid reduction of the mass effect on the optic chiasma (Muller et al. [17]).

In conclusion, the diagnosis of CP with febrile meningitis caused by the rupture of a cyst is unusual. This case highlights the importance of cerebral imaging on aseptic meningitis without a diagnosis, as well as the need to perform a precise anamnesis of the days and weeks before meningitis, which can reveal details about visual and endocrine disturbances. Lastly, a specific biochemical analysis of CSF helped explain the pathophysiology of the meningitis, in this case with evidence of cholesterol-rich content in the CSF.

## Data Availability

Data sharing is not applicable to this article, as no datasets were generated or analyzed during the current study.

## Abbreviations

CP: Craniopharyngioma
CSF: Cerebrospinal fluid
ACP: Adamantinomatous craniopharyngioma
PCP: Papillary craniopharyngioma
